# Technique of Gene Expression Profiles Extraction Based on the Complex Use of Clustering and Classification Methods

**DOI:** 10.3390/diagnostics10080584

**Published:** 2020-08-12

**Authors:** Sergii Babichev, Jiří Škvor

**Affiliations:** 1 Department of Informatics, Faculty of Science, Jan Evangelista Purkyně University in Ústí nad Labem, 40096 Ústí nad Labem, Czech Republic; Jiri.Skvor@ujep.cz; 2 Department of Computer Science, Software Engineering and Economic Cybernetics, Faculty of Computer Science, Physics and Mathematics, Kherson State University, Kherson 73003, Ukraine

**Keywords:** gene expression profiles, lung cancer, clustering, classification, ML-based binary classifiers, SOTA clustering algorithm, clustering quality criteria, ROC analysis, fuzzy inference system

## Abstract

In this paper, we present the results of the research concerning extraction of informative gene expression profiles from high-dimensional array of gene expressions considering the state of patients’ health using clustering method, ML-based binary classifiers and fuzzy inference system. Applying of the proposed stepwise procedure can allow us to extract the most informative genes taking into account both the subtypes of disease or state of the patient’s health for further reconstruction of gene regulatory networks based on the allocated genes and following simulation of the reconstructed models. We used the publicly available gene expressions data as the experimental ones which were obtained using DNA microarray experiments and contained two types of patients’ gene expression profiles—the patients with lung cancer tumor and healthy patients. The stepwise procedure of the data processing assumes the following steps—in the beginning, we reduce the number of genes by removing non-informative genes in terms of statistical criteria and Shannon entropy; then, we perform the stepwise hierarchical clustering of gene expression profiles at hierarchical levels from 1 to 10 using the SOTA (Self-Organizing Tree Algorithm) clustering algorithm with correlation distance metric. The quality of the obtained clustering was evaluated using the complex clustering quality criterion which is considered both the gene expression profiles distribution relative to center of the clusters where these gene expression profiles are allocated and the centers of the clusters distribution. The result of this stage execution was a selection of the optimal cluster at each of the hierarchical levels which corresponded to the minimum value of the quality criterion. At the next step, we have implemented a classification procedure of the examined objects using four well known binary classifiers—logistic regression, support-vector machine, decision trees and random forest classifier. The effectiveness of the appropriate technique was evaluated based on the use of ROC (Receiver Operating Characteristic) analysis using criteria, included as the components, the errors of both the first and the second kinds. The final decision concerning the extraction of the most informative subset of gene expression profiles was taken based on the use of the fuzzy inference system, the inputs of which are the results of the appropriate single classifiers operation and the output is the final solution concerning state of the patient’s health. To our mind, the implementation of the proposed stepwise procedure of the informative gene expression profiles extraction create the conditions for the increasing effectiveness of the further procedure of gene regulatory networks reconstruction and the following simulation of the reconstructed models considering the subtypes of the disease and/or state of the patient’s health.

## 1. Introduction

The use of gene expression datasets for the reconstruction of gene regulatory networks (GRN) and the simulation of the reconstructed models is one of the topical directions of current bioinformatics [1,2,3,4]. GRN in this case is a group of molecular elements interconnections that determines the functional possibilities of a biological organism. Qualitatively reconstructed GRN allows us to understand the particularities of genes interconnections and differences of these interconnections for healthy and ill cells, in order to create both new effective medicines and methods to treat complex diseases, such as Alzheimer’s, Parkinson’s, various types of cancer, and so forth. The results of both DNA microchip experiments and mRNA molecules sequencing methods are used to form the gene expression data nowadays [5,6]. In the first case, we have as a result the matrix of light intensities, the values of which are proportional to expression of appropriate gene (level of gene activity). Transformation of these light intensity into expression values assumes implementation of four steps—background correction [7,8,9,10], normalization [7,11,12,13,14,15], PM correction and summarization [7,13,16,17]. In the second case, the initial data is presented as a matrix of genes count, the values of which are varied in very wide range. In this case, the first step of the data processing involves a transform of this matrix into gene expression matrix using appropriate mathematical functions [18]. However, in any case, we receive as a result the high dimensional matrix of gene expressions, where quantity of genes is varied from 50–60 thousand genes. Under the gene expression profile in this case, we understand a set of gene expressions, the values of which are evaluated for various samples or under dissimilar conditions of the experiment carrying out. Each of the profile values corresponds to appropriate sample. Informative genes extraction in terms of the current problem is the first task which should be solved at the stage of the experimental data pre-processing. The informative genes extraction in this case means that it is necessary to extract mutually correlated gene expression profiles in terms of resolving ability of the studied samples (healthy and not-healthy patients or subtypes of disease). Biclustering technique is applied to solve this problem in the most cases nowadays [19,20,21]. Each of the biclusters contains a set of mutually correlated genes and samples. However, direct applying this technique to high-dimensional array of gene expressions leads to large number of biclusters and the choice from them the informative sets is very difficult and unsolved task nowadays. Moreover, in the most cases biclusters contains not complete set of samples. This fact also limits the range of the gene expression values’ variation during further simulation process.

The aforementioned presented facts indicate the relevance of the research concerning the extraction of groups of informative genes considering particularities of the investigated objects for purpose of further reconstruction of GRN based on the extracted genes and simulation of the reconstructed models. Within the framework of this research, we solve this problem based on the complex application of classification and clustering techniques with the use of fuzzy inference system at the final step of decision making concerning the extraction of a set of the informative gene expression profiles.

### 1.1. Problem Statement

The initial dataset is presented as a matrix of gene expressions: $\left(e_{ij}\right)\in R^{n\times m}$, where *n* and *m* are the number of samples and genes respectively. We suppose that the samples can be divided into previously known classes. The main problem consists of the extraction of genes, which allow us to divide the samples into classes maximally correctly in terms of the used criteria.

### 1.2. Literature Survey

There are a lot of works which are devoted to gene expression data processing nowadays. So, in Reference [22] the authors considered reducing the non-informative gene expression profiles using both Shannon entropy and statistical criteria. They supposed that gene expression profile can be removed from the data if its Shannon entropy value is larger and variance and average of absolute values are less in comparison with appropriate boundary values. To determine the boundary values the authors used fuzzy inference system and clustering quality criteria. The result of the proposed technique applying is removing genes which has zero or low expression values for all samples (lowly expressed genes), low level of gene expression variation for samples various types (do not allow distinguishing samples) and chaotic variation of the expression values for investigated samples (high value of Shannon entropy). In this study, we have applied the results of the authors research.

References [23,24] considered the issues concerning the bicluster analysis of gene expressions data. Implementation of this technique allows the extraction of groups of mutually correlated rows and columns. In Reference [23] the researchers presented an enhanced version of the Pearson’s correlation coefficient (PCC) to achieve better biclustering-enabled co-expression analysis. The obtained results were established both statistically and biologically using benchmarked gene expression data. In Reference [24] the authors proposed a novel approach for gene expression data biclustering with the use of fusion of differential evolution framework and self-organizing Kohonen’s map (SOM). The proposed approach was applied to two real-life microarray gene expression datasets and the obtained results were compared with various current techniques. References [25,26] present research results concerning the implementation of various clustering techniques for single-cell RNA sequencing data processing. Within the framework of the research, the authors carried out four experiments using two big scRNA-seq datasets with the use of twenty models. The obtained results allowed authors to conclude that the proposed feature extraction increased the quality of high-dimensional and sparse scRNA-seq data. The authors have also shown that the proposed feature-extraction techniques can promote to the clustering performance.

The issues concerning gene extraction to solve the problem of cancer types classification are considered in Reference [27]. The authors proposed a new hybrid wrapper procedure, the application of which allows the combining of the parameters of a teaching learning-based algorithm and a gravitational search algorithm. They have shown also that proposed technique is expressively outmatch existing metaheuristic methods relating to convergence rate, classification accuracy and optimal quantity of used features. A new multi-classification technique based on combining the probabilistic support vector machine and elastic net was described in Reference [28]. Applying this technique can solve the problem of cancer detection using gene expression profiles data of platelets. The authors applied within the framework of the research the probabilistic support vector machine in order to produce the outputs of the binary classifiers with class-specific features matching. The obtained results have shown that the presented technique is well-suited for traditional multi-classification tasks in the case using datasets with high-dimension of features and small quantity of samples.

In Reference [29], the authors proposed a new approach for semi-supervised classification of time-series. The proposed techniques learn both from labeled and unlabeled data. The authors have shown that the proposed approach approach substantially outperforms the state-of-the-art semi-supervised time-series classifier. The results of the research concerning the use of hubness-aware semi-supervised approach for classification of high dimensional gene expression data are presented in Reference [30]. The author proposed a self-training semi-supervised extension of Naive Hubness-Bayesian k-Nearest Neighbor. The author has also shown that the proposed approach can increase the classification accuracy and reduce computational costs. In Reference [31], the authors considered issues focused on the classification of gene expression data using extreme learning machines with regularization. The authors compared the proposed technique with different regularization strategies in context of a binary classification task related to gene expression data. Reference [32] presents the results of the research concerning development of non-invasive method of recognition of finger skin based on K-NN classifier. The authors have shown that the proposed approach can help us to diagnose pathologies of human skin.

However, we would like to note that accuracy of the classifier operation in the case of the use of high dimensional gene expression data depends on the vector of the extracted genes which are used as the classifier inputs. The perspective of our research is the reconstruction of a gene regulatory network based on the extracted genes and following simulation of the reconstructed models. In this case, the extraction of an optimal subset of gene expression profiles can increase the informativity of the reconstructed gene regulatory network and, as a result, it can create the conditions for better understanding of the character of genes’ interconnections during the following simulation process considering both the state of the patient’s health or subtype of disease. In Reference [33], we solve this problem based on stepwise application of clustering and biclustering techniques. Implementation of this procedure allowed us to remove gene expression profiles which were identified as noise using a density based DBSCAN clustering algorithm. Then, we divided the set of remaining genes into two subsets using SOTA clustering algorithm. At the final step, we applied the bicluster analysis to the obtained subset of gene expression profiles. To our mind, the main disadvantage of this technique is the following—the gene expression data were divided without considering the type of the used samples (state of the patients’ health or subtype of the disease). We used in this case only appropriate quantitative criteria. This fact can influence the quality of the reconstructed gene regulatory networks. This problem can be solved by using current techniques, models and information technologies, which are used successfully in various fields of scientific research nowadays [34,35]. Within the framework of this research, we propose the solution of this problem based on the complex use of clustering techniques, ensemble of binary classifiers and a fuzzy inference system using various quantitative quality criteria of both the clustering and classification procedures implementation.

The aim of this paper is the development of a technique of stepwise gene expression data extraction on the basis of complex use of cluster analysis, binary classifiers and fuzzy inference system. To our mind, it can contribute to increasing the objectivity of informative genes’ selection considering the state of the patients’ health for the purpose of both further gene regulatory networks’ reconstruction based on the allocated genes and simulation of the reconstructed models.

## 2. Materials and Methods

### 2.1. General Procedure of the Problem Solving

**Figure 1:**
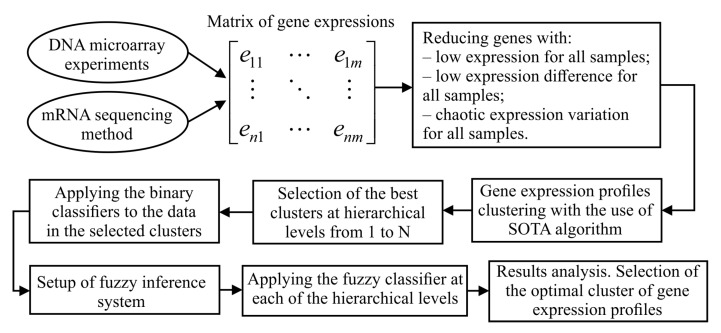
A structural chart of stepwise procedure of gene expression data processing.

Figure 1 shows the structure chart of stepwise procedure of gene expression data processing which was implemented within the framework of current research. As it can be seen from Figure 1, implementation of this procedure assumes solving the following tasks:

- formation of the matrix of gene expressions for the investigated samples. In the case of the use of DNA microarray experiments technique, this step involves background correction, normalization, PM correction and summarization. In the case of mRNA molecules sequencing method use, this step assumes allocation of genes count matrix and following transforming the values of this matrix into suitable range;
- extraction of genes which are identified as informative in terms of absolute value of gene expressions, variance and Shannon entropy.
- hierarchical clustering of gene expression profiles at the levels from 1 to N using SOTA clustering algorithm with correlation distance metric;
- division of the samples in allocated clusters of gene expression values into previously known classes and calculation of the quality criteria considering both the samples distribution within the appropriate classes and the distance between the samples in different classes;
- selection of the best clusters in terms of the used criteria at each of the hierarchical levels. These clusters correspond to the extreme values of the used quality criteria;
- applying the binary classifiers to the data in obtained clusters at each of the hierarchical levels. Formation of the intermediate solutions for each of the used classifiers and for each of the selected clusters;
- setup of fuzzy inference system. Definition of the membership functions for both the input and output variables, setup of ranges of the input and output parameters variation, knowledge base formation;
- applying the fuzzy classifier at each of the hierarchical levels;
- results analysis. Selection of the optimal cluster of gene expression profiles in terms of the used criteria.

### 2.2. Gene Expression Profiles Reducing

Implementation of this stage assumes removing genes, expressions profile of which were identified as non-informative in terms of variance, average of absolute values of gene expressions and Shannon entropy. We assumed that if gene expressions for all samples (averages of gene expressions are small) and differences of gene expression values for different samples (variances) are small, and if expression values for various samples are varied chaotically and this fact does not allow us to identify correctly the classes of the examined samples (Shannon entropy values are large), then, this gene can be removed from the dataset as non-informative one. The value of Shannon entropy for each of the gene expression profiles was calculated using James-Stein shrinkage estimator technique [36].

To evaluate the appropriate criteria boundary values, we apply the technique presented in Reference [22]. Applying this technique involves the following:

- calculation of variance, average of absolute values and Shannon entropy for each of the genes expression profiles. Formation of both the ranges of these criteria variation and steps of their values change;
- formation of clusters of the examined samples considering the data annotation. In the case of our dataset use, the samples can be divided into two clusters (with tumor and healthy samples);
- Determination of clustering quality criterion which is calculated at each step of change of the used criteria. Within the framework of our research, we used as the clustering quality criterion the multiplicative combination of WB-index [37] and Calinski Harabasz criterion [38]:
  (1) $$QC_{int}=\frac{QC_{WB}}{QC_{CH}}=\frac{K\left(K-1\right)QCW^{2}}{\left(N-K\right)QCB^{2}};$$
  where QCW and QCB are calculated as an average distance from objects to centers of the clusters where these objects are allocated and between centers of the clusters respectively:
  (2) $$QCW=\frac{1}{N}\sum_{s=1}^{K}\sum_{i=1}^{N_{s}}d\left(x_{i}^{s},C_{s}\right)$$
  (3) $$QCB=\frac{2}{K(K-1)}\sum_{i=1}^{K-1}\sum_{j=i+1}^{K}d\left(C_{i},C_{j}\right).$$
  Here, *K* is the clusters quantity; *N* is the number of samples; $N_{s}$ is the number of samples in the cluster *s*; $x_{i}^{s}$ is the *i*-th sample in the cluster *s*; $C_{i}$, $C_{j}$ and $C_{s}$ are the centers of the clusters *i*, *j* and *s* respectively; d(·) is the distance metric between vectors of gene expressions. Considering high dimension of the gene expressions vectors, we used the correlation distance as the distance metric. Minimum value of the criterion (1) corresponds to the optimal clustering;
- increasing the boundary values of variance and average of absolute values from minimum to maximum ones and Shannon entropy values from maximum to minimum one within the admissible ranges and removing genes for which the variance and average of absolute values are less and Shannon entropy is larger than appropriate boundary values. Calculation of the clustering quality criterion at each step of this procedure execution by the formula (1);
- result analysis. Fixation of the used criteria boundary values which correspond to minimum value of the clustering quality criterion;
- final removing the non-informative genes using determined boundary values of the statistical criteria and Shannon entropy.

Algorithm 1 presents the stepwise procedure of this stage implementation.
**Algorithm 1:** Gene expression profiles reducing.
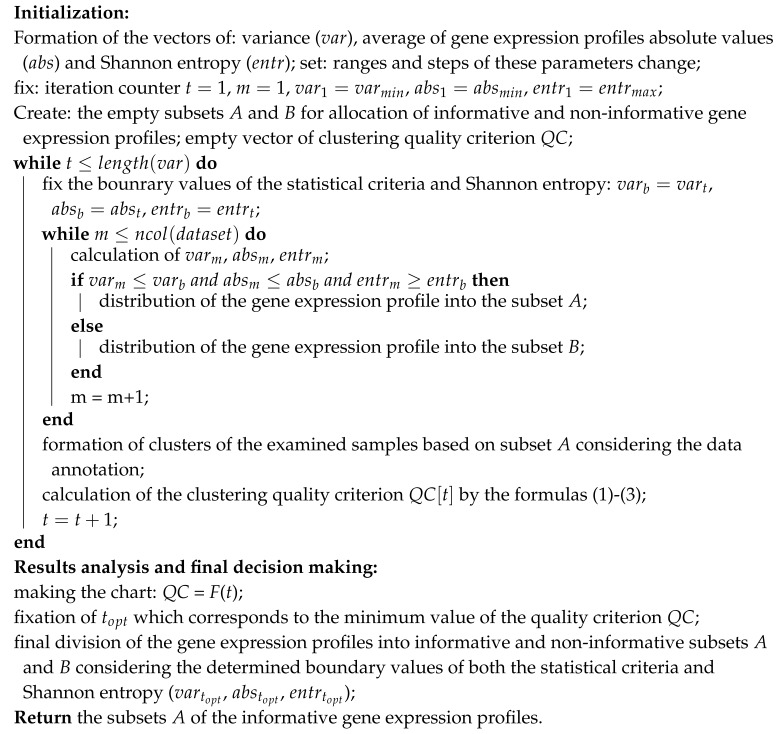


### 2.3. Stepwise Hierarchical Gene Expression Profiles Clustering

As was noted before, the main objective of this research is extraction of the most informative gene expression profiles in terms of their ability to identify the investigated samples considering both the state of the patient’s health or subtype of the disease. For this reason, the next stage of the previously presented procedure execution is stepwise gene expression profiles clustering at the hierarchical levels from 1 to *N*. We used the Self-Organizing Tree clustering Algorithm (SOTA) [39] with correlation distance metric for this step implementation. This algorithm is a variety of self-organizing neural networks and it is based on the complex apply of Kohonen maps and Fritzke algorithm of spatial cell structure growing [40]. The simulation results have shown that SOTA clustering algorithm with correlation distance metric divides the set of high dimensional gene expression profiles into two clusters at one step of this procedure execution [41]. Thus, the number of clusters is varied from 2 to $2^{N}$ at the first and the *N*-th hierarchical levels respectively. Then, we calculated the quality criterion values for each of the allocated clusters at each of the hierarchical levels using formulas (1)–(3). The vectors of genes expressions which correspond to the studied samples are used in this case as the investigated data. In other words, we evaluate in this case the proximity level of the samples, the attributes of which are the values of genes expressions which are grouped in the cluster. One cluster at each of the hierarchical levels was selected for the further research. These clusters correspond to the minimum value of the used quality criterion. Algorithm 2 for this stage implementation is presented below.
**Algorithm 2:** Stepwise hierarchical gene expression profiles clustering based on the use of Self-Organizing Tree clustering Algorithm (SOTA).
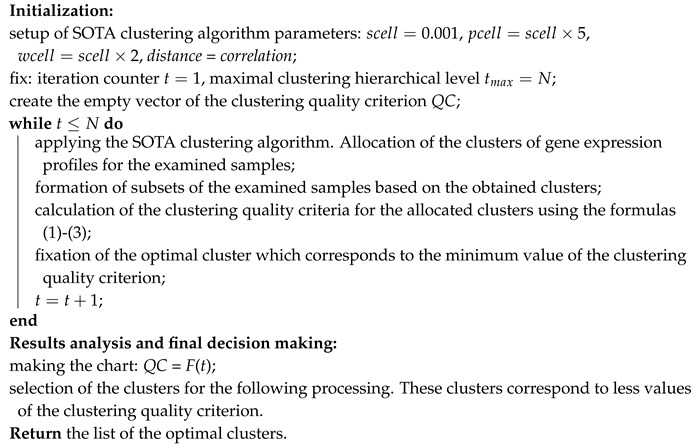


### 2.4. Binary Classification of the Investigated Samples

Four binary classifiers were used to evaluate the resolving ability of gene expression profiles in the selected clusters:

- Logistic Regression classifier (GLM) [42];
- Support-Vector Machine classifier (SVM) [43];
- Decision Tree classifier (CART) [44];
- Random Forest classifier (RF) [45].

The quality criteria based on the errors of both the first and the second kinds were used to evaluate the appropriate classifier effectiveness within the framework of the research. We used the gene expression data of patients, which were investigated on lung cancer disease. The data contained two types of samples—for healthy patients and patients with tumor. In this case, the classifier output can take two states: 0—healthy; 1—tumor. The obtained results in this case can be represented using a confusion matrix as follows (Table 1):

The following criteria were used to evaluate the classifiers effectiveness:

- *Accuracy* (AC) determines the total probability that classifier predicts true results:
  (4) $$AC=\frac{TP+TN}{TP+FP+TN+FN}.$$
- *F-measure* (*F*) is defined as a harmonic mean of *Precision* (PR - positive predicted values) and *Recall* (RC or Sensitivity) [46]:
  (5) $$F=\frac{2\cdot PR\cdot RC}{PR+RC}$$
  where:
  $$PR=\frac{TP}{TP+FP};\;\;\;RC=\frac{TP}{TP+FN}.$$
- *Matthews correlation coefficient* (MCC) used in machine learning as a measure of the quality of binary classifiers [47]:
  (6) $$MCC=\frac{\left(TP\cdot TN)-(FP\cdot FN\right)}{\sqrt{\left(TP+FP)\cdot (TP+FN)\cdot (TN+FP)\cdot (TN+FN\right)}}.$$
  Larger value of each of the criteria corresponds to higher classifier effectiveness.

### 2.5. Fuzzy Inference System Implementation

Necessity of the use of fuzzy inference system [48] is determined by the possible contradiction of the different classifier results for individual samples. To solve this problem, we propose to form the final solution using fuzzy inference system. Within the framework of our research the mathematical model can be presented as follows:

(7) $$FS=f(x_{GLM},x_{SVM},x_{CART},x_{RF}),$$

where FS is the output parameter of the fuzzy inference system characterized a final state of the investigated object (tumor or health); $x_{GLM},\;x_{SVM},\;x_{CART},\;x_{RF}$ are the input parameters or the results of GLM, SVM, CART and RF classifiers respectively. The values of both the input and output variables were varied within the range from 0 to 1. The fuzzy inference process executing assumes the following stages:

1. *Setup of the system*:
  - transforming the values of both the input and output variables into linguistic estimates. Formation of the membership functions for each of the variables;
  - formation of a basic term-set with appropriate membership function for each of the terms;
  - formation of a set of fuzzy rules which are agreed between input and output variables.
2. *Fazzification procedure*. This step assumes evaluation of the membership functions values for each of the input variables crisp values for each of the terms.
3. *Fuzzy inference process*. This step involves the following:
  - aggregation or determination of the conditions truth degree by clipping the levels for the prerequisites of each of the rules using the *min* operation;
  - activation or determining the truth degree for each of the fuzzy rules;
  - accumulation or forming the resulting membership function for output variable using *max* operation.
4. *Defuzzification* or determining the output variable crisp value.

### 2.6. Experiment

The publicly available gene expression data *GSE19188* of patients examined at the early stage of lung cancer [49] was used as the experimental data within the framework of the research. This dataset was obtained as a result of DNA microchip experiments and 156 DNA microchips were obtained during the experiment performance. The data annotation analysis has shown that the examined samples can be divided into two groups: 65 of the patients were healthy and 91 of the patients have lung cancer tumor. The *rma* method of data preprocessing (background correction, normalization, PM correction and summarization) was used to form the array of gene expression profiles. Initially, the data contained 54,675 of genes (maximum number of genes at each of the microchips). Thus, the initial dataset was formed as a matrix in size (156 × 54,675).

At the first step, the non-informative genes in terms of variance, Shannon entropy and average of absolute values were reduced in accordance with technique described hereinbefore in the Section 2.1. The simulation process assumed changing the boundary values of Shannon entropy from maximum to minimum value and appropriate statistical criteria values from minimum to maximum ones within the admissible ranges. Then, the gene expression profiles were identified as informative profiles for the following processing, if their average of absolute values and variance were larger and Shannon entropy was less than appropriate boundary values. Two clusters considering the state of the patients’ health were formed with following computation of the quality criterion by formulas (1)–(3) at each stage of this procedure execution.

## 3. Results and Discussion

**Figure 2:**
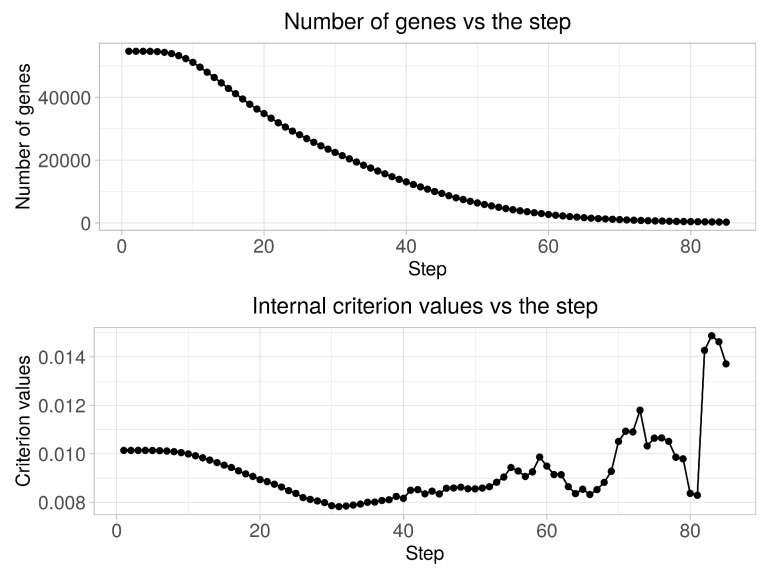
Diagrams of the number of genes in the clusters and the quality criterion values versus the step of the boundary parameters change.

Figure 2 presents the diagrams of both the quantity of genes in clusters and the clustering quality criterion values versus the step of the boundary parameters change. An analysis of the obtained charts allows the conclusion that the quality criterion achieves its minimum value at 31-st step. 21,431 of genes are identified in this case as informative ones. Thus, the initial matrix was transformed into matrix in size (156 × 21,431) as a result of this step implementation. At the next stage, we performed the stepwise gene expression profiles clustering at hierarchical levels from 1 to 10 using the SOTA clustering algorithm following the selection of the most informative groups of genes at each of the hierarchical levels in accordance with the technique described in Section 2.3. At the final step, we performed binary classification of the examined samples and carried out the fuzzy inference procedure for final solution making.

**Figure 3:**
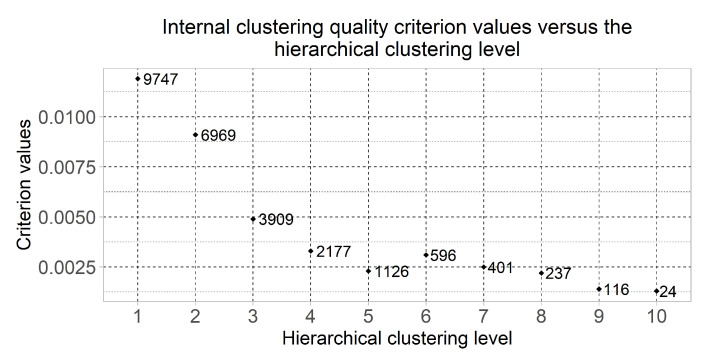
Dot plot of the clustering quality criterion calculated for the most informative clusters and the number of genes in this cluster versus the hierarchical clustering level.

Figure 3 displays the dot plot of the clustering quality criterion values of which were computed using the formulas (1)–(3) for the most informative clusters considering the minimum value of the quality criterion at each of the hierarchical levels. This chart also shows the number of genes in the selected clusters. The clusters quantity was changed from 2 to $2^{10}=1024$ at the first and the tenth hierarchical clustering levels respectively. Six of the clusters were selected for the following research as the result of the obtained chart analysis—the clusters which were allocated at hierarchical levels from 5 to 10. The cluster which was allocated at the fourth hierarchical level was not considered due to large quantity of genes.

The simulation process concerning examined samples classification was performed using “Caret” [50], “AER” [51], and “e1071” [52] packages of R software [53]. In the case of SVM classifier use, we used the “linear” kernel (this choice was done empirically. Considering the high dimension of the experimental data, the use of “radial” kernel gave significantly worse classification results). The optimal parameters “gamma” and “cost” were determined in each of the cases empirically using cross validation by the use of tune.svm() function of “e1071” package. The examined samples were divided into two subsets considering the class to which belong the appropriate samples. Sixty percent of samples contained data for the model treaning and the remaining 40% was used for testing process performance. In the case of logistic regression classifier (GLM) apply, we used glm() function with $family=binomial(link=\prime \prime logit^{\prime \prime })$. Decision tree and random forest classifiers were implemented based on “caret” package ising train() function. In both cases, we used 10 estimators.

Table 2, Table 3, Table 4 and Table 5 present the simulation results concerning application of *GLM*, *SVM*, *CART* and *RF* binary classifiers to classify the data in the selected clusters. The tables contain the results of the test datasets classification using previously trained classifiers.

**Figure 4:**
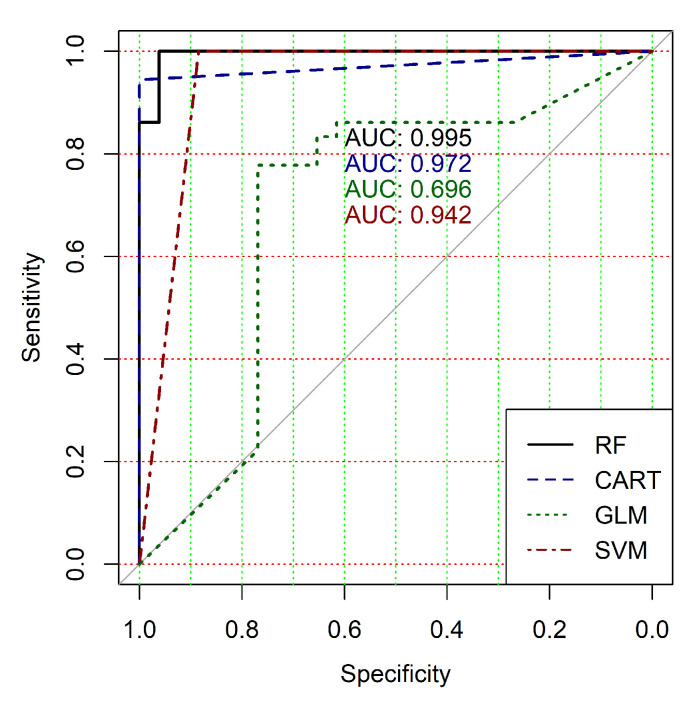
ROC curves for models of ML-based binary classifiers at the hierarchical clustering level 5.

**Figure 5:**
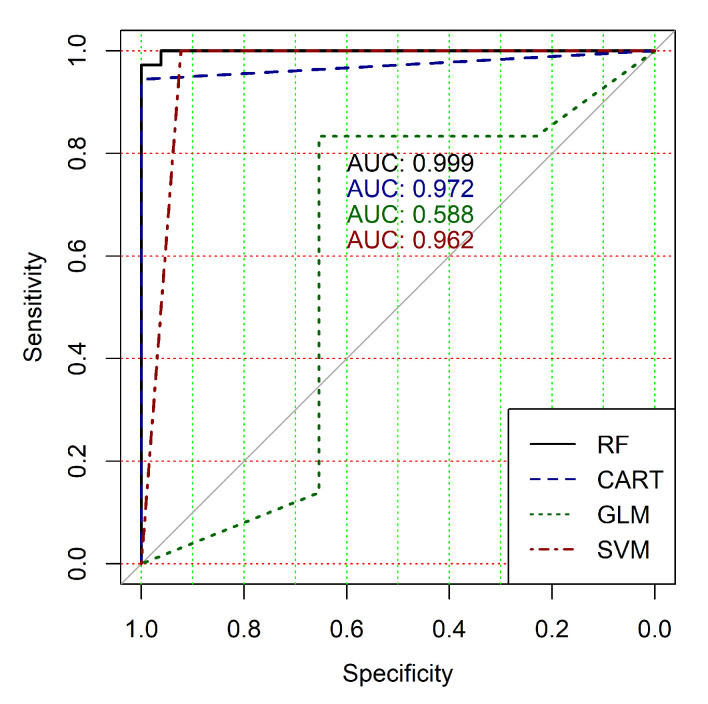
ROC curves for models of ML-based binary classifiers at the hierarchical clustering level 6.

**Figure 6:**
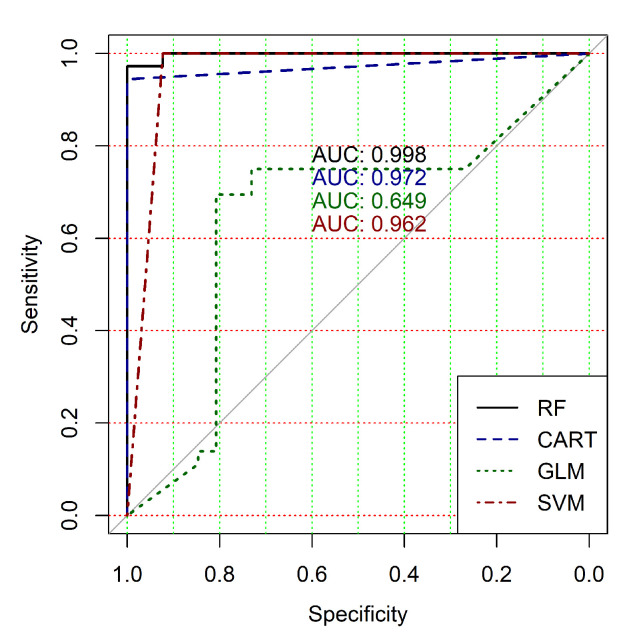
ROC curves for models of ML-based binary classifiers at the hierarchical clustering level 7.

**Figure 7:**
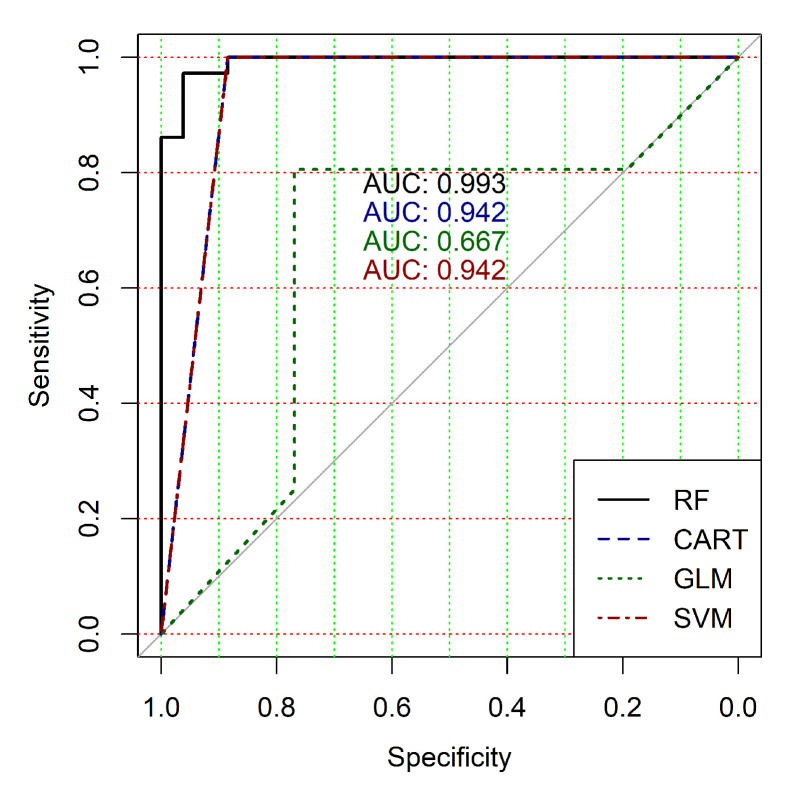
ROC curves for models of ML-based binary classifiers at the hierarchical clustering level 8.

**Figure 8:**
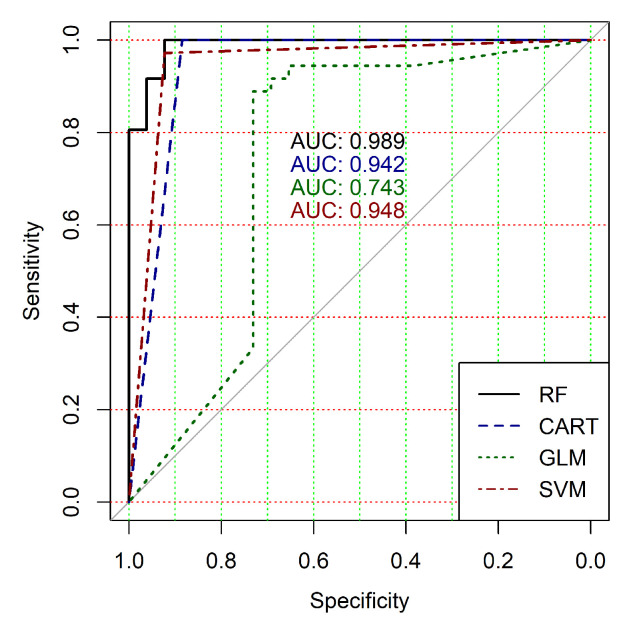
ROC curves for models of ML-based binary classifiers at the hierarchical clustering level 9.

**Figure 9:**
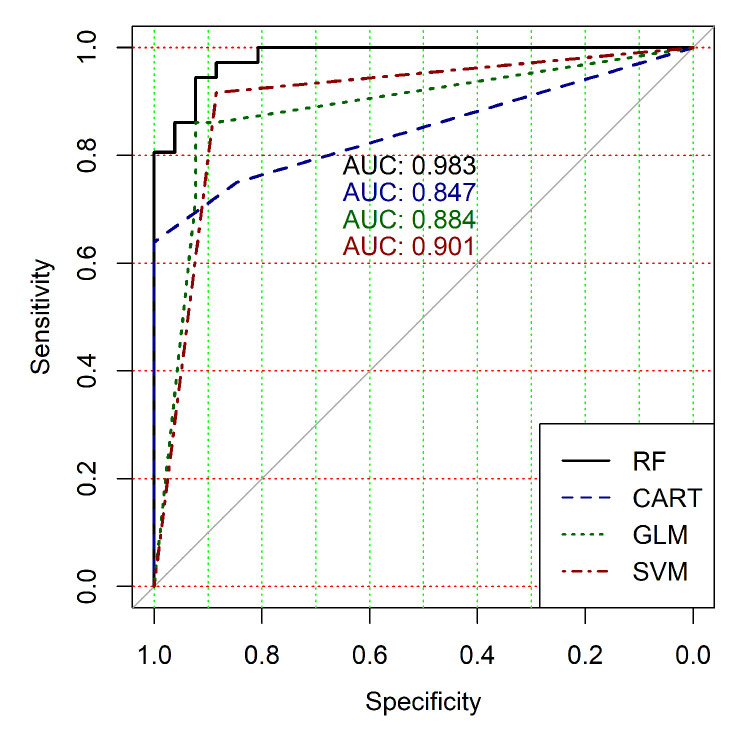
ROC curves for models of ML-based binary classifiers at the hierarchical clustering level 10.

The obtained results analysis allows the conclusion that a classifier based on the logistic regression model (GLM) is not effective for processing high-dimensional vectors of gene expressions. The classification results are not satisfactory in all cases. A little better result in terms of the used criteria was obtained in the case of the use of cluster, which was allocated at the tenth hierarchical level. This cluster contained only 24 of genes. However, the use of this classifier is not reasonable in the case of gene expression data classification. Significantly better results were obtained in the cases of other binary classifiers’ application. It should be noted that all classifiers show worse classification results in the case of the use of data in the smallest cluster (24 of genes). In other cases, the results of the classifications almost agree under the use of SVM, CART and RF classifiers. Some better results were obtained in the case of CART and RF classifiers use in comparison with the use of SVM classifier. Figure 4, Figure 5, Figure 6, Figure 7, Figure 8 and Figure 9 show the ROC curves of classification results for datasets allocated at hierarchical clustering levels from 5 (1126 of genes) to 10 (24 of genes) in the case of the use of all ML-based binary classifiers.

The analysis of the ROC curves confirms the conclusion concerning the low effectiveness of the GLM classifier (areas under the curves are 0.696, 0.588, 0.649, 0.667, 0.743 and 0.884 for clusters obtained at hierarchical levels from 5 to 10 respectively) and high effectiveness of RF, CART and SVM classifiers (areas under the roc-curves are significantly larger in comparison with areas obtained using GLM classifier). For this reason, we will use only the results of RF, CART and SVM classifiers as the input parameters of the fuzzy inference system at the next step of the simulation process.

We defined the terms “Healthy” (HL) and “Tumor” (TM) for input variables (result of appropriate classifier operation) and we used trapezoidal membership function for each of the terms. For output variable “Final State” (FS), we defined the terms: “Healthy” (HL); “Probably Healthy” (PHL); “Probably Tumor” (PTM); and “Tumor” (TM). We used also trapezoidal membership function for terms HL and TM and triangular membership function for terms PHL and PTM respectively.

**Figure 10:**
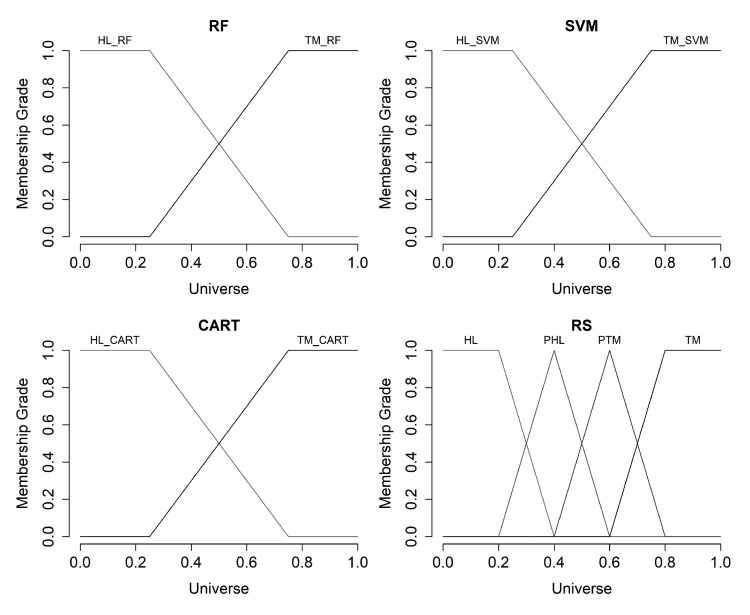
The charts of the membership functions for input and output variables.

Figure 10 shows the charts of the hereinbefore defined membership functions for input and output variables.

Table 6 presents the various combinations of the terms values which were used during the fuzzy rules formation. We applied Mamdani inference algorithm for fuzzy inference procedure performing and centroid method (mass center of the resulting membership function) for implementation of the defuzzification process.

Table 7 presents the results of fuzzy inference system operation.

The obtained results analysis allows us to conclude that in the case of fuzzy inference system use, we get some worse results for clusters obtained at hierarchical levels from 5 to 9 and a significantly better result for clusters obtained at hierarchical level 10. Moreover, an analysis of the classification result for clusters at the ninth hierarchical level shows disagreement of various binary classifiers applied in the previous step of our research despite very good classification results in the case of binary classifiers’ application. This fact indicates that the use of this cluster is not reasonable for the following research. Moreover, the complex analysis of both Figure 4 and Table 3, Table 4 and Table 5 and Table 7 indicates the reasonability of use for the further research the cluster obtained at hierarchical level 7. The cluster contains 401 gene expression profiles, the values of the clustering quality criterion are not large too, and classification results in terms of the used quality criteria are suitable in the case of the use of both separate binary classifiers and a hybrid model based on a fuzzy inference system.

**Figure 11:**
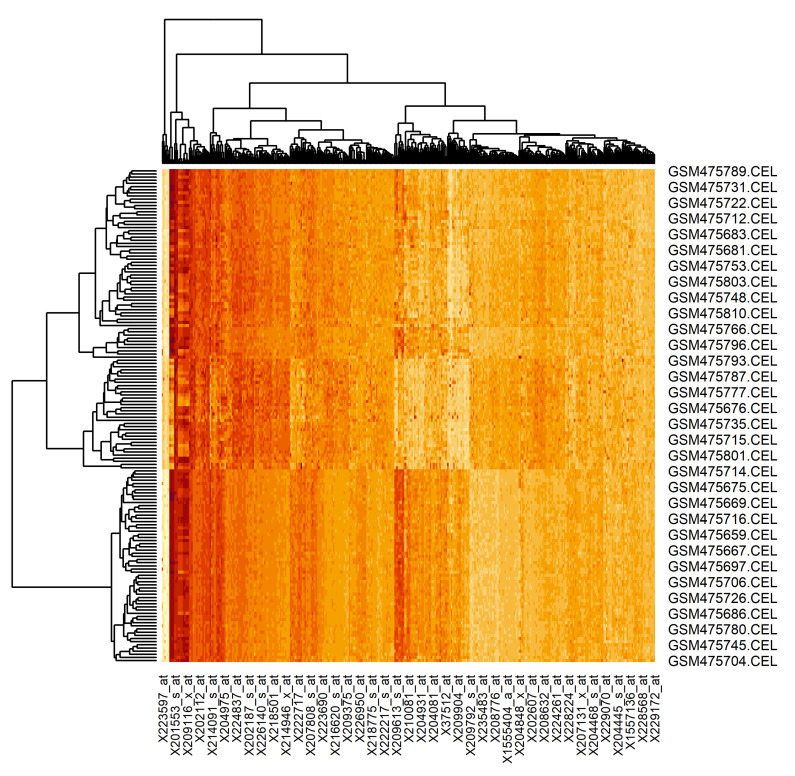
Heat map of the extracted gene expressions data (rows are the examined samples and columns are extracted genes)

The results of the proposed technique application is presented in Figure 11.

Here, the rows are the examined samples (156 in total, 65 of the patients are healthy and 91 of the patients have lung cancer tumor) and the columns are the extracted genes (401 of genes). As can be seen, the extracted genes really allow the division of the samples into two groups (result of the dendrogram analysis). Moreover, the samples of the patients with a lung cancer tumor can also be divided into subsets. It is naturally, since the state of the patients’ health in this case can be different too. Thus, the gene regulatory network, reconstructed based on the genes extracted using the proposed technique, can allow us to understand both the particularities of the genes’ interconnection and the influences of these interconnections on the state of the patients’ health.

## 4. Conclusions

In this paper, we have presented the results of the research concerning the extraction of a set of informative gene expression profiles in terms of their mutual correlation, based on the complex use of both the clustering and classification techniques. The initial data have been presented as a matrix of gene expressions $\left(e_{ij}\right)\in R^{n\times m}$, where *n* and *m* are the number of samples and genes respectively. The publicly available gene expression data *GSE19188* of patients examined at the early stage of lung cancer disease were used as the experimental data. This data contained 156 DNA microchips. The annotation of the data has shown that the examined samples can be divided into two groups—65 of the samples for healthy patients and 91 of the samples belong to patients with lung cancer tumors. The initial dataset contained 54,675 genes (maximal quantity of genes at DNA microchips).

As the first step, we extracted the informative gene expression profiles by removing low-informative genes in terms of statistical criteria and Shannon entropy. In this case, we have used the clustering quality criterion as the main measure to evaluate the boundary values of the appropriate criteria. The initial matrix was transformed into a matrix of a size of (156 × 21,431) as a result of this step implementation. At the next stage, we performed the step-by-step gene expression profiles clustering at hierarchical levels from 1 to 10 with the use of the SOTA clustering algorithm, following the selection of the most informative clusters in terms of the used clustering quality criterion at each of the hierarchical levels. The number of clusters was changed from 2 to $2^{10}=1024$ at the first and at the tenth hierarchical clustering levels respectively. Six of the clusters were selected for the following research as the result of this step implementation—the clusters that were allocated at hierarchical levels from 5 to 10.

Then, we carried out the classification of the examined samples using four well known binary classifiers—Logistic regression classifier (GLM); Support-vector machine classifier (SVM); Decision tree classifier (CART); Random forest classifier (*RF*). The quality criteria based on errors of both the first and the second kinds have been used to evaluate the appropriate classifier effectiveness. The analysis of the obtained results has shown that classifier based on logistic regression model is not effective to process the high-dimensional vectors of gene expressions. Significantly better results have been obtained in the cases of other binary classifiers applying. However, it should be noted that all classifiers have shown worse classification results in the case of the use of data in the smallest cluster (24 of genes). In other cases, the results of the classifications almost agree under the use of SVM, CART and RF classifiers. Some better results have been obtained in the case of CART and RF classifier use in comparison with the use of the SVM classifier. The simulation results have also shown that some of the examined samples were identified differently and applying the fuzzy classifier to increase the objectivity of the gene expression profiles’ extraction at the final step is reasonable.

The analysis of the results of the fuzzy inference system operation allows the conclusion that we have some worse results for clusters obtained at hierarchical levels from 5 to 9 and a significantly better result for clusters obtained at the 10th hierarchical level. Moreover, an analysis of the classification result for clusters at the 9th hierarchical level has shown disagreement of various binary classifiers despite very good classification results in the case of the use of individual classifiers. This fact indicates that the use of this cluster is not reasonable for the following research. The analysis of the obtained results has also shown the reasonability of using the cluster obtained at hierarchical level 7 for further research. This cluster contains 401 genes, the value of the clustering quality criterion is not large, and the classification results in terms of the used quality criteria are suitable in terms of both separate binary classifiers and the hybrid model based on the fuzzy inference system.

The results of the proposed technique application has been presented using the heat map, where the rows and columns are the examined samples and the extracted genes respectively. The analysis of the heat map has confirmed the fact that the extracted genes really allow the division of the samples into two groups. Moreover, the samples of the patients with lung cancer tumors can also be divided into subsets considering the state of the patients’ health. Thus, the gene regulatory network was reconstructed based on the genes extracted using the proposed technique, which allows us to understand both the particularities of the genes’ interconnection and the influences of these interconnections to the state of the patients’ health.

To our mind, the conducted research can allow us to increase the objectivity for the extraction of genes, which can be used for the reconstruction of gene regulatory networks and the simulation of the reconstructed models considering the subtype of disease and/or state of the patient’s health. Further, we are going to use the obtained results for both the gene regulatory networks reconstruction based on allocated genes and the simulation of the reconstructed models in order to better understand the gene interconnection in the cases of various states of the patient’s health. This is the perspective of our research.

## Figures and Tables

**Table 1:** Confusion matrix for lung cancer disease diagnostic.

Real State of Test-Objects	Testing Result	
	Tumor Predicted	Norm Predicted (Healthy)
Tumor (1)	True positives (TP)	False negatives (FN)
Healthy (0)	False positives (FP)	True negatives (TN)

**Table 2:** Results of logistic regression classifier operation (GLM).

Hierarchical Level	Quality Criteria				
	AC	PR	RC	F	MCC
5	0.543	0.474	0.450	0.462	0.066
6	0.565	0.526	0.476	0.500	0.118
7	0.565	0.632	0.480	0.545	0.148
8	0.522	0.579	0.440	0.500	0.060
9	0.587	0.579	0.500	0.537	0.169
10	0.804	0.895	0.708	0.791	0.629

**Table 3:** Results of support-vector machine classifier operation (SVM).

Hierarchical Level	Quality Criteria				
	AC	PR	RC	F	MCC
5	0.913	0.842	0.941	0.889	0.821
6	0.935	0.895	0.944	0.919	0.865
7	0.935	0.895	0.944	0.919	0.865
8	0.913	0.842	0.941	0.889	0.821
9	0.913	0.895	0.895	0.895	0.821
10	0.848	0.842	0.800	0.821	0.689

**Table 4:** Results of decision tree classifier operation (CART).

Hierarchical Level	Quality Criteria				
	AC	PR	RC	F	MCC
5	0.968	1.000	0.929	0.963	0.936
6	0.968	1.000	0.929	0.963	0.936
7	0.968	1.000	0.929	0.963	0.936
8	0.952	0.885	1.000	0.939	0.904
9	0.952	0.885	1.000	0.939	0.904
10	0.790	0.846	0.710	0.772	0.588

**Table 5:** Results of random forest classifier operation (RF).

Hierarchical Level	Quality Criteria				
	AC	PR	RC	F	MCC
5	0.952	0.885	1.000	0.939	0.904
6	0.968	0.923	1.000	0.960	0.935
7	0.968	0.923	1.000	0.960	0.935
8	0.952	0.885	1.000	0.939	0.904
9	0.968	0.923	1.000	0.960	0.935
10	0.903	0.923	0.857	0.889	0.805

**Table 6:** Terms values of the input and output variables.

Number of Fuzzy Rules	Input and Output Variables			
	$x_{\mathrm{SVM}}$	$x_{\mathrm{CART}}$	$x_{\mathrm{RF}}$	FS
rule 1	*T*	*T*	*T*	*T*
rule 2	*T*	*T*	*H*	PT
rule 3	*T*	*H*	*T*	PT
rule 4	*H*	*T*	*T*	PT
rule 5	*H*	*H*	*T*	PH
rule 6	*H*	*T*	*H*	PH
rule 7	*T*	*H*	*H*	PH
rule 8	*H*	*H*	*H*	*H*

**Table 7:** Results of fuzzy inference system operation.

Hierarchical Level	Quality Criteria				
	AC	PR	RC	F	MCC
5	0.913	0.842	0.941	0.889	0.821
6	0.935	0.895	0.944	0.919	0.865
7	0.935	0.895	0.944	0.919	0.865
8	0.935	0.895	0.944	0.919	0.865
9	0.891	0.842	0.889	0.865	0.775
10	0.913	0.895	0.895	0.895	0.821

## Abbreviations

The following abbreviations are used in this manuscript:

DNA	Deoxyribonucleic Acid
RNA	Ribonucleic Acid
SOTA	Self-Organizing Tree Algorithm
ROC	Receiver Operating Characteristic
PM	Perfect-Match
GRN	Gene Regulatory Network
PCC	Pearson’s Correlation Coefficient
GLM	Generalized Linear Model
SVM	Support-Vector Machine
CART	Classification And Regression Trees
RF	Random Forest
